# Tai-Chi for Residential Patients with Schizophrenia on Movement Coordination, Negative Symptoms, and Functioning: A Pilot Randomized Controlled Trial

**DOI:** 10.1155/2012/923925

**Published:** 2012-11-24

**Authors:** Rainbow T. H. Ho, Friendly S. W. Au Yeung, Phyllis H. Y. Lo, Kit Ying Law, Kelvin O. K. Wong, Irene K. M. Cheung, Siu Man Ng

**Affiliations:** ^1^Department of Social Work and Social Administration, The University of Hong Kong, Hong Kong; ^2^Centre on Behavioral Health, The University of Hong Kong, Hong Kong; ^3^The Hong Kong Sheng Kung Hui Providence Garden for Rehab, Hong Kong

## Abstract

*Objective*. Patients with schizophrenia residing at institutions often suffer from negative symptoms, motor, and functional impairments more severe than their noninstitutionalized counterparts. Tai-chi emphasizes body relaxation, alertness, and movement coordination with benefits to balance, focus, and stress relief. This pilot study explored the efficacy of Tai-chi on movement coordination, negative symptoms, and functioning disabilities towards schizophrenia. *Methods*. A randomized waitlist control design was adopted, where participants were randomized to receive either the 6-week Tai-chi program and standard residential care or only the latter. 30 Chinese patients with schizophrenia were recruited from a rehabilitation residency. All were assessed on movement coordination, negative symptoms, and functional disabilities at baseline, following intervention and 6 weeks after intervention. *Results*. Tai-chi buffered from deteriorations in movement coordination and interpersonal functioning, the latter with sustained effectiveness 6 weeks after the class was ended. Controls showed marked deteriorations in those areas. The Tai-chi group also experienced fewer disruptions to life activities at the 6-week maintenance. There was no significant improvement in negative symptoms after Tai-chi. *Conclusions*. This study demonstrated encouraging benefits of Tai-chi in preventing deteriorations in movement coordination and interpersonal functioning for residential patients with schizophrenia. The ease of implementation facilitates promotion at institutional psychiatric services.

## 1. Introduction

Schizophrenia affects about 24 million people worldwide. Despite a low incidence rate (3/10,000) [1, 2], its poor recovery prognosis and chronic nature renders it a highly prevalent disorder (0.4%–1%). Long-term care and symptoms management become crucial as symptoms may persist lifelong. While mild-graded patients under medication may live independently, others may benefit from residential care which prepares them for reentering the community when their symptoms and personal care, are well-managed. 

Under the medical model, the primary focus of care and research on patients prioritizes illness management, self-care, and functioning abilities while physical or psychological well-being falls secondary. Patients with schizophrenia have comparatively shorter life expectancies due to poor physical health (higher incidence of cardiovascular and metabolic diseases) and psychological health (depression and suicide) [3–5]. However, this may be partially attributable to the side-effects of medication; poor lifestyles or a simple lack of exercise [6]. 

There are a number of known benefits of exercise to patients' psychosocial well-being and their symptoms of psychiatric disorders. Levin and Gimino [7] indicated that aerobic exercise reduces depression, anxiety, and obsessive-compulsive symptoms in hospitalized schizophrenic patients compared to no-exercise controls. Similar interventions improved mood, anxiety and depression, increased self-esteem, energy, concentration, quality of life, and social interactions [8, 9]. Faulkner and Sparkes [10] investigated a 10-week exercise program which reduced auditory hallucinations, raised self-esteem, improved sleep patterns, and general behaviors. Randomized controlled trials established even stronger evidence of aerobic and strength exercises in lowering positive and negative symptoms, state anxiety, and psychological distress while improving quality of life [11]. More recent research noted anatomical changes associated with aerobic exercise particularly in increasing hippocampal volume [12] which potentially improves short-term memory among exercisers with schizophrenia. Besides symptom-related outcomes, a lack of physical activity was associated with worse health-related quality of life [13].

Exercises based upon Eastern health philosophy like Tai-chi stress the interrelated body and mind. Besides being a form of light-to-moderate intensity physical exercise [14, 15] which improves cardiovascular fitness, balance control, and flexibility [16], Tai-chi is also a cultivation of psychological focus and relaxation [17]. There is strong empirical evidence on the mind-body effects of Tai-chi for the elderly, depressed patients and those suffering from coronary heart diseases [18, 19]. Besides symptom-specific improvements, regular practice of Tai-chi can also effectively enhance physical and mental quality of life for various patient populations [20, 21]. Through a decrease in the neuroendocrinal stress response, it brings about psychological benefits for chronic patients through its antidepressant and antiolytic effects [22]. Tai-chi is principled upon body relaxation, mental alertness, movement sequencing, and coordination [23]. Targeting both the mind and body, Tai-chi holds promising benefits for patients with mental illnesses. A randomized-controlled trial [24] on a 12-week Tai-chi program for patients with chronic schizophrenia found reduced negative symptoms. Another form of mind-body intervention, yoga, which also stresses breathing, relaxation and stretching, was found effective in reducing positive and negative symptoms while enhancing health-related quality of life in a systematic review of randomized-controlled trials [25]. Emphasizing focus cultivation, relaxation, bodily coordination, and control, Tai-chi can potentially improve psychopathogical symptoms, movement coordination, and general functioning. However, little research investigated the potential benefits of Tai-chi for these clienteles.

The present study explored the effectiveness and implementation feasibility of Tai-chi (Wu-style Cheng form) on the movement coordination, negative schizophrenic symptoms, and general functioning disabilities for residential schizophrenic patients. This pilot trial was conducted for patients with schizophrenia residing at residential rehabilitation facilities. Halfway houses and long-stay care homes offer training on illness management and life skills so as to facilitate community reintegration [26]. This reflects a gradual departure from relying purely on medical treatment to incorporating adjuvant nonpharmalogical interventions. Yet, institutionalized patients with schizophrenia, particularly those in long-stay care homes, suffer from higher cognitive impairments, more serious negative symptoms and worse social functioning compared to their counterparts in other living conditions [27]. If the benefits of Tai-chi can be established, this can be a promising contribution to residential mental illness rehabilitation. Not only does it facilitate illness management, the mastery of a Tai-chi movement sequence may further promote independence and a sense of control over illness outcomes. 

The primary aim of this pilot trial is to examine the effectiveness of a 6-week Tai chi program as an adjunctive intervention in a residential rehabilitation setting. The secondary objective is to explore the benefits and disadvantages of such intervention, thereby, informing the feasibility for promotion and areas for improvement.

## 2. Methods

### 2.1. Subjects

The pilot trial recruited thirty residential patients with chronic schizophrenia from the Sheng Kung Hui Providence Garden for Rehab, a mental health rehabilitation complex in Hong Kong providing both long-stay care and halfway house services to enhance the heterogeneity of the sample. Potential participants were invited to participate by their social workers based on the following inclusion and exclusion criteria. 30 participants were recruited so as to ensure an optimal group size of 12 after randomization [28] and a dropout rate of 20%.

The inclusion criteria included the following. (a) A diagnosis of schizophrenia according to the DSM IV-TR criteria. (b) Age between 18 to 65 years. (c) Ability to understand and speak Cantonese. (d) no prior experience in learning Tai-chi. Participants were excluded if (e) diagnosed with acute schizophrenia requiring hospitalization; (f) suffering from severe schizophrenic symptoms (e.g., persistent withdrawal) that would limit their ability to interact or participate in the class; (g) suffering from physical disabilities that would limit Tai-chi practice (including past or current serious spinal, hip or knee injury or pathology; severe pain limiting movement; or unsuitable for Tai-chi exercise as determined by their physicians); (h) Suffering from other severe illnesses which may impair cognitive or visuomotor functioning, cause physical pain or limit life expectancy to 10 years or less.

Ethical approval for this study was granted by the Human Research Ethics Committee for Non-Clinical Faculties at the University of Hong Kong. Written informed consent was solicited from all participants.

### 2.2. Intervention and Waitlist Randomization

This Tai-chi class was based on the Wu-style Cheng-form Tai-chi Chuan [29] led by mental health professionals. They were provided formal training in Tai-chi before going through a 12-session Tai-chi trainer's course at a professional Tai-chi institute. While movement is relatively standardized across various styles of Tai-chi, the unique strengths of the Wu-style (Cheng form) Tai-chi are its emphasis on movement rhythm, with potential benefits on movement coordination. It comprises of 22 simple movement forms (listed in Table 1) which are relatively easy and emphasizes attention and coordination in their basic philosophy [29]. One is required to name the movement form during practice which demands attention, concentration, memory and physical exertion inclusive in one simple exercise routine. One-hour classes were held twice weekly for 6 weeks with 15 participants. Additional half-hour trainer-led practice sessions were held on a weekly basis throughout the 12-week study period, accumulating to a total of 2.5 hours of Tai-chi practice per week.

Randomization of participants was done using random numbers. The waitlist group received their standard residential care which includes a 30-minute daily morning stretching routine for both the Tai-chi and control participants.

### 2.3. Measurements

Both the Tai-chi and waitlist groups were assessed (i) before intervention (T1), (ii) within 1 week after the 6-week intervention (after intervention T2), and (iii) within the 6th week after the intervention (maintenance T3). Qualitative feedback on learning and practising Tai-chi were collected with a structured interview schedule at T3 on their perceived benefits and difficulties of practising Tai-chi. Responses were recorded on paper by the interviewer.

A series of patient assessments were administered by trained research assistants who were blinded to the group assignment of participants.

#### 2.3.1. Movement Coordination Tests

Measurements of arm-hand dexterity and rapid eye-hand coordination were assessed using the Minnesota Rate of Manipulation Test (CMDT) [30], which is a collection of structured and well-established tests on motor disabilities used for occupational planning. It consists of five sub-tests: placing, turning, displacing, one-hand turning/placing, and two-hand turning/placing. Scoring is based on time needed to complete the tasks which involve the manipulation of cylindrical discs on two boards with perforated holes. A practice trial was given for all tests followed by two trials of which the average score was taken. Higher scores indicate longer time needed to complete the task, hence greater disabilities in movement coordination. The CMDT has good test-retest reliability for psychiatric patients (including schizophrenia) [31] and is currently being used as a formal motor assessment at the test site.

#### 2.3.2. Scale for the Assessment of Negative Symptoms (SANSs)

The SANS [32] was used for the assessment of negative symptoms in 5 dimensions including attention, anhedonia-asociality, avolition-apathy, alogia, and affective flattening or blunting. The scale is rated on a 6-point Likert scale where higher scores indicate greater severity of negative symptoms.

To ensure inter-rater reliability, the first 5 interviews were conducted with multiple interviewers and final ratings were given after deliberation by the team. The rated scores would subsequently be discussed with psychiatric social workers providing care to the participants to ensure that ratings given closely reflected their actual symptom levels.

#### 2.3.3. World Health Organization Disability Assessment Schedule (WHODAS-II)

The WHODAS-II [33] measures levels of health-induced disabilities in a number of life domains in the past 30 days. Domains include cognition, mobility, self-care, interpersonal interactions, life activities, and community participation. The internal consistency, test-retest reliability, and validities of this instrument were satisfactory for patients with schizophrenia [34, 35]. 

The interviewer-administered 36-item version was adopted. Items were translated and back-translated by the research team into Chinese to facilitate participants' understanding. Certain items including “staying by yourself for a few days” and “sexual activities” were dropped for a lack of relevance at the studied residential facility. Higher scores denote greater functioning disabilities.

#### 2.3.4. Sociodemographic and Clinical Information

Patients' sociodemographic and clinical information were solicited from personal and medical records. This included their age, gender, education level, martial status, and employment. Their period of psychiatric diagnosis, medication, and consecutive lengths of stay at residential facilities were collected as clinical data. 

#### 2.3.5. Qualitative Feedback

Structured interview questions requiring participants to list their subjective advantages and disadvantages of Tai-chi were posed at the maintenance assessment. 

### 2.4. Statistical Analyses

Intention-to-treat analysis was employed, such that participants with missed Tai-chi sessions or data were still included in the final analysis. Due to the small sample size, nonparametric techniques were adopted. Within and between-group comparisons were conducted with the Wilcoxon signed ranks the Mann-Whitney *U* tests, respectively, using the Statistical Package for Social Sciences version 18.0. Analyses were conducted on all three time points for intervention and maintenance effects. All statistical significance tests are two-sided, at the level of significance of *P* ≤ 0.05. Effects sizes were calculated according to [36], where medium and large effects sizes are indicated by *r* = 0.3 and *r* = 0.5, respectively. Missing variables were replaced with the median of the respective subscale if the number of missing variables did not exceed half of the subscale. Qualitative feedback was analyzed using theme analysis.

## 3. Results

### 3.1. Participants

**Figure 1:**
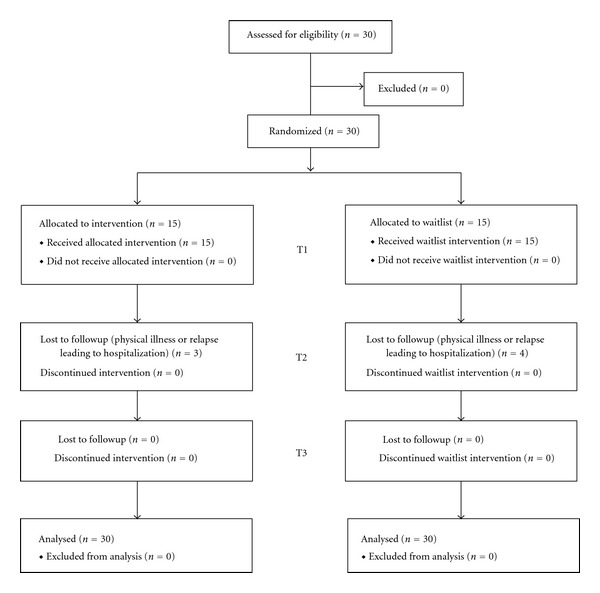
Participant flowchart.

30 participants fulfilling the inclusion and exclusion criteria were invited to participate into the study (Figure 1).

Participants had a mean age of 53 years and were diagnosed for about 28 years. The average consecutive length of stay at rehabilitation residencies was 11.8 years. All participants were receiving antipsychotic medication.

On Chi-square and Mann-Whitney tests, the Tai-chi and the waitlist groups were comparable on their sociodemographic and clinical statues as well as the assessment variables (Table 2). The sole exception is a relatively higher ratio of females in the waitlist group as compared to the Tai-chi group.

Antipsychotic medication use at baseline (T1) did not differ significantly between the two groups which were assessed based on chlorpromazine equivalents which reflects medication dosage (*Z* = −0.591; *P* = 0.555) [37]. The average daily chlorpromazine equivalents of the Tai-chi and waitlist groups were 391 mg and 365 mg, respectively. Medication change at maintenance (T3) was minimal, where only two participants from the Tai-chi group and one from the waitlist group had their dosages altered during the study period. The average change in the chlorpromazine equivalents did not significantly differ between the groups (*Z* = −0.537; *P* = 0.592). Approximately 57% of the participants (*n* = 17) were taking atypical antipsychotics.

### 3.2. Movement Coordination

Motor dexterity and eye hand coordination (CMDT) after attending the Tai-chi class (T2) was not vastly different from baseline (T1). But the waitlist group experienced significant deterioration on 3 of the 5 tests of the CMDT, the turning test (*Z* = 2.22; *P* = 0.026; *r* = 0.57), the displacing test (*Z* = 2.22; *P* = 0.026; *r* = 0.57) and the one-hand test (*Z* = −2.98; *P* = 0.003; *r* = 0.77). 

There is a significant difference in how the Tai-chi group and the waitlist group faired on the displacing test (*Z* = −2.28; *P* = 0.023; *r* = 0.42) and marginal significance the one-hand test (*Z* = −1.95; *P* = 0.065; *r* = 0.36). Therefore, the Tai-chi class buffered from deteriorations in movement coordination but effects were not sustained at maintenance (T3).

### 3.3. Negative Symptoms

Changes to negative symptoms were not statistically significant after the Tai-chi class or in the waitlist group. Between group comparison also failed to reach significance. 

### 3.4. Functioning Disability

Fewer disruptions in life activities functioning was observed for the Tai-chi group at maintenance (T3) (*Z* = −2.14; *P* = 0.03; *r* = 0.55). The Tai-chi participants also found fewer difficulties with community participation at T2 (*Z* = −2.73; *P* = 0.01; *r* = 0.70). The waitlist group, however, experienced greater disruptions in interpersonal functioning at T2 (*Z* = −2.22; *P* = 0.03; *r* = 0.57) and sustained at T3 (*Z* = −2.43; *P* = 0.02; *r* = 0.63). Between group differences in interpersonal functioning were marginally significant between baseline and T2 (*Z* = −2.56; *P* = 0.07; *r* = 0.47) and significant between baseline and T3 (*Z* = −2.56; *P* = 0.01; *r* = 0.47). Performance outcomes of the two groups are detailed in Table 3.

### 3.5. Qualitative Feedback on the Benefits and Disadvantages of Tai-Chi

Participants generally enjoyed Tai-chi for the benefit it brought to their physical and mental health. Others found it to be a pleasurable activity although a few did not enjoy the level of persistence required by the slow yet energy-demanding Tai-chi movements. Other difficulties arose from the complexity of movements.  Table 4 lists the themes and examples of the feedback.

## 4. Discussion and Conclusion

Tai-chi is often taken as a form of alternative therapy in the treatment for physical or mental ailments [17, 38]. In schizophrenia research, the possible benefits of this traditional form of mind-body exercise have not been receiving similar attention as other types of physical exercises. The benefits of physical activity to the rehabilitation of psychosis are well established although patients tend to be less physically active compared to those without psychosis [39]. Poor physical fitness, low skeletal-muscle mass, and obesity, all of which are associated with a lack of exercise, are all contributing factors to mortality among patients with schizophrenia [40].

The purpose of this study was to demonstrate that the detrimental manifestations of schizophrenia are amendable by lifestyle modification like regularly practicing Tai-chi. Results lent evidence that Tai-chi can help protect against deteriorations in movement coordination after 6 weeks of Tai-chi. With regular weekly practice, it also buffered against a decline in interpersonal functioning which was sustained even 6 weeks after the class. Reasons for the deteriorations in the waitlist group may reflect the instability of psychomotor or functioning states of residential psychiatric patients. The majority of participants required long-stay care, with unstable illness prognosis and functioning. Particularly since the collection of T2 and T3 data happened to fall during festivities, participants' daily functioning and activities may have been affected by family visits, or other activities held at the facility.

Previous exercise interventions for schizophrenia rehabilitation focused primarily on the alleviation of psychotic symptoms rather than psychomotor outcomes, despite being an important illness manifestation. The current pilot trial showed how movement coordination can experience pronounced benefits after exercising. Tai-chi emphasizes movement rhythm, which may have helped prevent motor deteriorations as a result of schizophrenia or extra-pyramidal symptoms. This outcome concurs with research demonstrating improved motor responses and postural control among the elderly regularly practising Tai-chi [41]. For patients with schizophrenia, better psychomotor functioning is related to social, clinical, and functioning outcomes [42]. Motor functioning is a key feature of schizophrenia and can be tied in with a number of other psychological symptoms. Hallucination, for instance, was found to be related to the blood flow to the motor region of the brain [43] leading to growing interest in the way mental events control movements. This, among other evidence, demonstrates a sophisticated interaction between psychological and motor processes in patients. Mind-body exercises like Tai-chi not only restore muscular strength and coordination but further cultivate psychological focus and concentration [17]. Given the intricate associations between the mental and motor processes in schizophrenia, the benefits Tai-chi has on the psychological states of patients may possibly underlie one of the pathways to better motor functioning. While this pathway has yet to be attested in future research, the buffering effect Tai-chi demonstrated on motor deteriorations holds important clinical implications. In residential settings, movement coordination can possibly help sustain self-care abilities, the completion of daily tasks, while indirectly supporting participation in social activities. Among participants in the current study, the general level of movement coordination on all five tests was substantially impaired, falling within the 1st percentile of the population norm [44]. Their performance was also largely confounded by deficiencies in understanding instructions. Therefore, despite promising outcomes, a more simple coordination test may more reliably reflect movement coordination function in future research.

Exercise-related benefits to interpersonal functioning are particularly relevant to group exercises like Tai-chi. Despite focusing on the inner self [17], traditional Tai-chi practice often takes place in a group, under a belief that stronger *qi* (a positive healing force) can be better cultivated in a group than by a single person alone. Therefore, the Tai-chi class allows for both verbal and nonverbal connections among participants. Another study on yoga intervention for patients with schizophrenia proposed the role of improved emotional recognition to enhancing social functioning [45]. Being socially integrated is especially important in residential settings, where many participants in the study complained of being emotionally affected by indifferent or disruptive relationships with fellow residents. Indeed, social functioning is a much neglected aspect of adjunctive treatment outcomes that cannot be captured by psychopathological assessments alone [46]. Yet, it bears significant clinical relevance where interpersonal interactions and community participation are predictive of clinical outcomes in a high risk psychosis group [47].

Participant feedback provided anecdotal suggestions for the possible mechanisms of the benefits of Tai-chi. Similar to yoga, Tai-chi distinguishes itself from other forms of physical exercises. Recognizing the mind-body nature of this activity, some participants expressed appreciation for both physical and mental benefits. They experienced improvements in health, flexibility, assured of the benefits for bones and ligaments. On the cognitive and psychological level, some participants were happier, more relaxed, alert, thinking more openly, and feeling more regulated. A hallmark disability of schizophrenia is poor learning and memory, believed to arise from hippocampal atrophy. With a high cognitive component involving the memorization of movement sequence, it can possibly help instigate hippocampal neurogenesis, hence, leading to cognitive improvements [48]. Future studies could also expand understanding on the benefits of Tai-chi towards other functional arenas.

From this preliminary study, Tai-chi supplementing regular antipsychotic and rehabilitation care has a protective effect for institutionalized patients. It is also a sustainable form of treatment that may offer a sense of mastery towards illness control. A persistent obstacle is participants' low motivation to continue practising independently. Tai-chi calls for mental endurance and patience, which proved challenging for some who found it mundane, or experience difficulty remembering the movements. Consequently, trainer-led weekly practice sessions could be helpful. Outside residential facilities however, practice sessions may become less feasible in the community, where preintervention psychoeducation on the health benefits of exercise and enhancing self-efficacy may improve participation rates [49]. In addition, this study was conducted with participants with constrained lifestyles where diet, sleep, medication, exercise levels, smoking, amongst others were carefully controlled. Under such favourable conditions, the effectiveness of Tai-chi is maximal, as it often takes collaborative efforts in lifestyle changes to bring about improvements to illness symptoms. Consequently, the effectiveness of Tai-chi for community patients with a less healthy lifestyle has yet to be explored.

Despite numerous studies demonstrating the effects of exercising and Tai-chi, this is one of the few randomized controlled trials on Tai-chi for patients with schizophrenia. However, this remains a small-sampled pilot study, which is confounded by the lack of a group exercise control condition to account for the possible effects of exercise or peer gathering. Another limitation lies in the assessment of functioning disability, WHODAS-II which was translated to Chinese but has yet to be validated in the population. Two items were also removed from the scale for their irrelevancy to the context but should also be assessed in a larger study with both residential and community patients. Notwithstanding such limitations, Tai-chi proved promising in areas where psychotropic medication currently has limited effectiveness. As the rehabilitation of mental illness gradually moves away from the medical model, Tai-chi can be promoted as an adjunct to improving patients' general functioning.

## Figures and Tables

**Table 1:** The 22 movement forms of Wu-style Cheng form Tai-chi.

Tai-chi (Wu-style Cheng form) movements	
(1) Ready style (*預備式*)	
(2) Tai chi beginning style (*太極起式*)	
(3) Seven stars style (*七星勢*)	
(4) Grasping a bird's tail (*攬雀尾*)	
(5) Single whip (*單鞭*)	
(6) Gliding diagonally (*斜飛勢*)	
(7) Raising hands and stepping up (*提手上勢*)	
(8) Flapping wings (*白鶴亮翅*)	
(9) Brush knee and twist step (*摟膝拗步*)	
(10) The seven stars style (Left) (*左* *七星勢*)	
(11) Brush knee and twist step (*摟膝拗步*)	
(12) The seven stars style (Left) (*左* *七星勢*)	
(13) Playing the lute (*手揮琵琶*)	
(14) Step up, parry and punch (*上步搬攔捶*)	
(15) Door shutting motion (*如封似閉*)	
(16) Embrace tiger and return to mountain (*抱虎歸山*)	
(17) Crossing hands (*十字手*)	
(18) Diagonally brush knee and twist step (*斜* *摟膝拗步*)	
(19) Turn body, brush knee, and twist step (*轉* *身* *摟膝拗步*)	
(20) The seven stars style (*七星勢*)	
(21) Grasping a Bird's tail (*攬雀尾*)	
(22) Diagonal single whip (*斜* *單鞭*)	

**Table 2:** Socio-demographical, clinical characteristics and assessment outcomes at baseline.

Variables	Tai-chi Mean (SD)	Waitlist Control Mean (SD)	P*
Age	51.87 (10.85)	53.47 (8.63)	0.69

Gender			0.03*
Male	9	3	
Female	6	12	

Education level			0.47
No formal education	3	4	
Primary	3	7	
Lower secondary (Grades 7–9)	3	2	
Upper secondary (Grades 10–11)	4	2	
After secondary or above	1	0	
Missing	1	0	

Marital status			0.94
Single	10	9	
Married	1	2	
Divorced/Separated	3	3	
Widowed	1	1	

Employment			0.52
Full time employment	0	1	
Unemployed	4	9	
Retired	11	5	
Years of diagnosis	29.47 (14.86)	26.2 (10.09)	0.33
Length of stay at residencies (years)	12.87 (14.82)	10.73 (9.87)	0.45
Chlorpromazine equivalent (mg)	391.07 (472.25)	365.12 (221.92)	0.85

*Assessment outcomes *	Tai-chi	Waitlist Control	P*

Movement Coordination (CMDT)			
Placing	82.17 (9.75)	98.29 (28.2)	0.12
Turning	76.73 (14.51)	101.04 (40.89)	0.10
Displacing	60.6 (10.13)	76.07 (26.61)	0.11
One-Hand Turning/Placing	102.37 (17)	124.82 (45.95)	0.17
Two-Hand Turning/Placing	65.53 (10.74)	89.96 (43)	0.16

Negative symptoms (SANS)			
Attention	4.27 (3.39)	5.6 (3.81)	0.21
Anhedonia-asociality	4.53 (4.29)	4.4 (4.6)	0.80
Avolition-apathy	1.67 (2.82)	2.27 (3.37)	0.79
Alogia	4.4 (4.97)	4.8 (5.17)	0.83
Affective flattening or blunting	8.2 (7.98)	8.67 (7.76)	0.68

Functioning disabilities (WHODAS II**)			
Cognition	12.2 (3.45)	11.33 (5.34)	0.31
Mobility	7.53 (2.39)	7.6 (2.87)	0.93
Self-care	3.4 (0.83)	3.2 (0.41)	0.61
Interpersonal interactions	7.47 (4.16)	5.27 (2.25)	0.08
Life activities	11 (3.61)	9.4 (1.76)	0.15
Community participation	17 (5.84)	14.73 (5.87)	0.38

**Table 3:** Performances outcomes of the Tai-chi and the waitlist control group on the assessed variables.

							Between group interaction	
Assessment outcomes	Tai-chi (*n* = 15)			Waitlist control (*n* = 15)			PostGroup effect T1-T2	Maintenance effect T1–T3
	T1	T2	T3	T1	T2	T3	*P*	*P*
Movement Coordination (CMDT)								
Placing	82.17 (9.75)	85.42 (16.78)	81.5 (14.63)	98.29 (28.2)	114.13 (41.04)	111.27 (45.09)	0.17	0.24
Turning	76.73 (14.51)	81.71 (20.6)	76 (17.4)	101.04 (40.89)	122.92 (50.61)*	115.88 (50.22)	0.11	0.15
Displacing	60.6 (10.13)	59.21 (12.13)	58.83 (9.78)	76.07 (26.6)	81.25 (26.57)*	84.23 (38.13)	0.02*	0.50
1-Hand Turning/Placing	102.37 (17)	105 (20.45)	101.53 (15.61)	124.82 (45.95)	145.33 (62.87)**	147.92 (74.8)	0.07	0.15
2-Hand Turning/Placing	65.53 (10.74)	66.25 (17.49)	67.07 (14.25)	89.96 (43)	101.09 (44.5)	107.31 (68.32)	0.17	0.26

Negative symptoms (SANS)								
Attention	4.21 (3.51)	2.67 (7.27)	3.33 (3.33)	5.6 (3.81)	3.55 (2.2)	5.85 (4.28)	0.06	0.49
Anhedonia-asociality	4.53 (4.29)	3.58 (3.63)	3.13 (2.45)	4.4 (4.6)	6 (4.07)	3.4 (3.09)	0.24	0.63
Avolition-apathy	1.79 (2.89)	1.58 (2.02)	1.79 (2.36)	2.27 (3.37)	3.27 (3.35)	2.14 (3.28)	0.62	0.84
Alogia	4.4 (4.97)	2.08 (4.12)	3.27 (5.46)	4.8 (5.17)	6.91 (4.81)	6.8 (7.29)	0.06	0.15
Affective flattening/blunting	8.2 (7.98)	3.33 (6.21)	6.4 (7.34)	8.67 (7.76)	7.82 (6.75)	6.47 (8.27)	0.56	0.59

Functioning disabilities (WHODAS II***)								
Cognition	12.2 (3.45)	12.27 (4.86)	11.13 (4.85)	11.33 (5.34)	9.87 (2.9)	11.4 (5.46)	0.93	0.44
Mobility	7.53 (2.39)	7.47 (3.42)	8.73 (4.17)	7.6 (2.87)	6.6 (2.06)	7.73 (2.63)	0.75	0.67
Self-care	3.4 (0.83)	3.07 (0.26)	3.47 (1.06)	3.2 (0.41)	3.27 (1.03)	3.73 (1.33)	0.41	0.33
Interpersonal interactions	7.47 (4.16)	7.13 (2.59)	6.13 (2.07)	5.27 (2.25)	7 (2.39)*	7.2 (3.1)*	0.07	0.01**
Life activities	11 (3.61)	10.27 (4.01)	9.47 (3.44)*	9.4 (1.76)	10.53 (4.36)	8.8 (1.82)	0.21	0.64
Community participation	17 (5.84)	12.87 (4.12)**	14.27 (6.27)	14.73 (5.87)	12.4 (4.03)	13.53 (6.33)	0.42	0.33

T1: baseline; T2: after-intervention; T3: 6-week maintenance; *P ≤ .05; ** P ≤ .01. ***Slightly modified.

**Table 4:** Themes and selected quotes on the subjective advantages and disadvantages of Tai-chi.

Advantages of Tai-chi	Disadvantages of Tai-chi
(1) Improving physical well-being, flexibility and movement regulation	(1) Tiredness
Tai-Chi was good for my bones and ligaments	Classes were long and felt out of energy
Tai-Chi made me more flexible	(2) Bodily discomfort
I was able to regulate the rhythm	My arms and legs hurt and I felt dizzy
It improved my physical ability	(3) Difficulty of the Tai-chi movements
It made me healthier	Movements were hard to remember and follow
(2) Improving cognitive and psychological health	(4) Difficulty in practicing independently
It made me happier	I did not know how to practice by myself
It helped me relax	(5) Tai-chi being slow and mundane
I could think more openly	It was boring
I felt more alert	
(3) Possibility of becoming a leisure activity	
Tai-Chi was attractive	
It gave me something to do	
(4) Others	
It was the correct thing to do	
Tai-Chi was a form of exercise	
